# Prevalence and Determinants of Anemia and Iron Deficiency in Kuwait

**DOI:** 10.3390/ijerph120809036

**Published:** 2015-07-31

**Authors:** Sameer Al Zenki, Husam Alomirah, Suad Al Hooti, Nawal Al Hamad, Robert T. Jackson, Aravinda Rao, Nasser Al Jahmah, Ina’am Al Obaid, Jameela Al Ghanim, Mona Al Somaie, Sahar Zaghloul, Amani Al Othman

**Affiliations:** 1 Kuwait Institute for Scientific Research, P.O. Box 24885, Safat 13109, Kuwait; E-Mails: omirah@kisr.edu.kw (H.A.); suad.alhooti@gmail.com (S.A.H.); jsager@kisr.edu.kw (J.A.G.); aothman@kisr.edu.kw (A.A.O.); 2 Food and Nutrition Administration, Ministry of Health, P.O. Box 24225, Safat 13103, Kuwait; E-Mails: nawal.alhamad@gmail.com (N.A.H.); m.alsumaie@gmail.com (M.A.S.); 3 Department of Nutrition and Food Science, University of Maryland, College Park, MD 20740, USA; E-Mail: bojack53@gmail.com; 4 Ministry of Health, Medical Laboratories Services, Sabah Hospital Laboratories, P.O. Box 24225, Safat 13103, Kuwait; E-Mails: aravindarao@hotmail.com (A.R.); nasseraljahmah@yahoo.com (N.A.J.); inaobz218@hotmail.com (I.A.O.); 5 National Nutrition Institute, 16 Kasr El Aini Street, P.O Box11311, Cairo, Egypt; E-Mail: zaghloulsahar@gmail.com

**Keywords:** epidemiology, public health, RBC folate, vitamin A

## Abstract

The objective of this study was to assess the prevalence of anemia and iron deficiency (ID) of a nationally representative sample of the Kuwait population. We also determined if anemia differed by socioeconomic status or by RBC folate and vitamins A and B12 levels. The subjects who were made up of 1830 males and females between the ages of 2 months to 86 years, were divided into the following age groups (0–5, 5–11, 12–14, 15–19, 20–49, ≥50 years). Results showed that the prevalence of anemia was 3% in adult males and 17% in females. The prevalence of ID varied according to age between 4% (≥50 years) and 21% (5–11 years) and 9% (12–14 years) and 23% (15–19 years), respectively, in males and females. The prevalence of anemia and ID was higher in females compared to males. Adults with normal ferritin level, but with low RBC folate and vitamins A and B12 levels had higher prevalence of anemia than those with normal RBC folate and vitamins A and B12 levels. This first nationally representative nutrition and health survey in Kuwait indicated that anemia and ID are prevalent and ID contributes significantly to anemia prevalence.

## 1. Introduction

Anemia is defined as a public health condition in which the number of red blood cells is insufficient to meet the body’s physiologic needs with iron deficiency (ID) being the predominant nutrient deficiency causing anemia [1]. Anemia and ID are associated with several adverse sequelae, including low birth weight, increased perinatal and maternal mortality, decreased immunocompetence, decreased work productivity, and decreased school performance [2,3]. Deficiencies of other nutrients (including folate, vitamin B12, and vitamin A) may contribute to anemia [4,5]. Information on the prevalence and etiology of anemia is essential to gauge the scope of anemia and also as a baseline against which to evaluate future interventions aimed at eliminating anemia.

Most studies of anemia in Kuwait have focused on the genetic causes of anemia such as sickle cell disease or thalassemia [6,7]. Estimates of nutritional causes of anemia have been based on small epidemiological studies, or clinical, or hospital-based studies [8,9,10] with limited generality. Past studies in Kuwait revealed that anemia is prevalent and may affect significant numbers of people across the life cycle [11,12,13,14]. There are few published studies on ID [14,15]. Recently, 50% prevalence of ID was found in a group of Kuwaiti college women [16].

Kuwait is a small country (1.3 million citizens) divided into 6 regions, called governorates. Development of oil brought dramatic changes to living standards, including lifestyle changes (especially decreased physical activity and increased food and energy intakes), diet changes associated with the proliferation of fast food restaurants [17,18,19]. The purposes of this study were to provide national prevalence estimates of anemia and ID and to establish baselines for future interventions and studies of anemia in Kuwait.

## 2. Experimental Section

### 2.1. Sampling Design

A cross-sectional, household based cluster survey of a representative sample of Kuwaiti nationals drawn from the six governorates of the State of Kuwait was carried out. In this sampling frame, Kuwait has been divided into 2000 clusters units with 20 to 300 or more households in each cluster. To ensure a “proportionate to population size” or PPS sampling method, the percentage of Kuwaiti households and total number of households in these clusters were utilized as the primary indicators for stratifying the population of Kuwait into 54 strata. From each cluster, 20 households were selected using stratified sampling. At the household level, subjects were randomly selected from each age category taking into consideration census gender distribution. This sampling frame was based on the most recent 2005 Kuwait census population [20].

### 2.2. Population

Eighty-two clusters were sampled from all of Kuwait in proportion to population size. The final sample size was of 1830 individuals from 545 families living in separate households. These included males and females between the ages of 2 months and 86 years, divided into age groups (0–5, 5–11, 12–14, 15–19, 20–49, and ≥50 years of age) The survey was conducted between November 2008 and December 2009 and included data gathered by interviews on to structured questionnaires, physical measurements, and measurements of biochemical variables (including hemoglobin (Hb), serum ferritin (SF), erythrocyte zinc protoporphyrin (ZnPP), serum retinol, red blood cell (RBC) folate, vitamin B12, and dietary investigation. The dietary investigation included dietary data that was collected using 24 h recalls and dietary histories. The 24 h recall data were collected from participants older than 2 years of age. The dietary history was used for participants younger than 2 years old. Proxy respondents were permitted for participants younger than 5 years of age, and 5–11 year participants were assisted by care takers. The 24 h recall questionnaire was designed to capture food and beverage consumed during the last 24 h to estimate total intake of food energy (calories), nutrients, and non-nutrient food components. Questions were added to describe overall participant’s diet. The questions included participant’s perception of his or her intake whether usual intake, more or less than usual, plain water consumption, frequency of added salt at the table, and supplement usage. Survey protocols were approved by the Ministry of Health, and adults gave their informed written consent for themselves or for their children. Data were collected from 7 primary health care clinics. The survey collected a variety of socio-demographic information (age, gender, educational level, marital status, income, *etc.*).

### 2.3. Blood Analysis

Fasting blood samples were collected after an overnight fasting by trained phlebotomists and were analyzed at the Reference Laboratory of the Ministry of Health. For children 5 years old or less, 1 mL of blood was collected in Ethylene Diamine-Tectra Acetic (EDTA) Microtainer tube for Complete Blood Count (CBC) analysis. For the age groups more than 5 years of age; SF, RBC folate, and vitamin B12 were assayed on Cobas e601 immunoassay analyzer (Roche Diagnostics, Boston, MA, USA) using manufacturer’s kits. ZnPP was assayed in the washed cells of whole blood samples using a portable AVIV hematoflurometer (AVIV Biomedical, Lakewood, NJ, USA). CBC (including RBC, hemoglobin) was performed on a Cell Dyne 4000 hematology analyzer (Abbott Laboratories, Irving, TX, USA). Serum retinol was determined using reverse phase high performance liquid chromatography (HPLC) according to the laboratory protocol for NHANES 2003–2004 [21].

Anemia was defined according to the WHO cutoffs: blood Hb < 110 g/L for children less than 5 years old, <115 g/L for the 5 to 11-years old males and females; <120 g/L for 12 to 14yearsold males and females; <120 g/L for females 15 years old and older-; and <130 g/L for males 15 years old and older [2]. ID was defined as an SF value of < 15 μg/L [2] for those more than 5 years old. The cut-offs for low vitamin A, RBC folate, and vitamin B12 were <30 μg/dL, <140 ng/mL and <200 pg/mL, respectively [22,23].

### 2.4. Data Analysis

Data were analyzed using SAS (version 9.2, SAS Institute, Cary, NC, USA). Due to the complex sampling design, sample weights were calculated and used in the data analysis to obtain national estimates of parameters (e.g., Hb, SF). Sample weights, primary sampling units, and stratification variables were considered such that differential probabilities of selection, adjustments for noncoverage and nonresponse bias could be accounted for [24,25]. National estimates were made using SAS survey procedures, using calculated weights. Regression analysis was also performed using SAS survey methods. A significant result was taken to be *p* < 0.05.

## 3. Results

### 3.1. Prevalence of Anemia among the Various Age Groups

Table 1 shows Hb concentrations and prevalence of anemia by age and gender groups. For males, the prevalence of anemia was highest in the youngest age groups, but declined with age. For females, anemia prevalence was high, at the youngest and oldest groups. Ten percent (10%) of males and 12% of females between the ages 5 and 11 years old were anemic. Conversely, the prevalence of anemia in the younger adolescent males and females (12–14 years) were higher in males (9%), as opposed to females (6%). For males and females 15 to 19 years old, anemia was more prevalent in females (10.2%) than in males (2.4%). A similar observation was also noted for young males and females 20 to 49 years old. Females had a significantly higher (*p* < 0.001) prevalence of anemia (17.1%) than males (2.5%). The mean Hb concentration of adult males 50 years and older was lower than that of younger adult males. Moreover, the percent of older adult males who were anemic was higher. For older females (≥50 years), the percent with anemia (18%) exceeded the percent in males (5.5%).It is noteworthy to mention that Hb concentrations of subjects did not differ by income, marital status, or levels of educational attainment for males or females (*p* > 0.05).

**Table 1:** Mean Kuwaiti Hb concentration and percent anemic by gender and age group.

Age(y)	n	Males		Females	
		Mean ± SEM *	% Anemic	Mean ± SEM *	% Anemic
0–4.9	252	123.9 ± 1.6	15.6	122.8 ± 2.0	15.7
5–11	181	131.7 ± 0.9	9.9	130.6 ± 1.1	12.4
12–14	182	140.9 ± 2.2	8.8	132.5 ± 1.8	5.7
15–19	194	152.8 ± 1.7	2.4	131.0 ± 1.4	10.2
20–49	663	156.6 ± 1.1	2.5	127.2 ± 1.0	17.1
≥50	358	149.1 ± 1.4	5.5	128.0 ± 0.9	18.0

* SEM = standard error of the mean.

### 3.2. Prevalence of Iron Deficiency among the Various Age Groups

Table 2 shows the prevalence of ID among various age groups of both genders based on SF level of <15 μg/L. The ID percentage declined with age for males, but not for females. The prevalence of ID among the 5 to 11 years old was similar for males and females; while it was higher for males than females among the 12 to 14 years old (11% *vs.* 9%). Among the 15 to 19 years old, ID was significantly more prevalent in females (22.6%) than males (4.2%). Similar results were found among adult females of 20 to 49 years old and 50 years and older. However, the prevalence of ID (by SF) was slightly less in older adult females, compared to younger adult females (15% *vs.* 19%).

**Table 2:** Prevalence of iron deficiency * by age and gender in Kuwaitis.

Age (y)	Males		Females	
	SF *	ZnPP **	SF	ZnPP
0–4.9	ND ***	1.9	ND	1.1
5–11	21.4	15.9	20.7	17.0
12–14	11.4	27.9	8.6	26.0
15–19	4.2	15.1	22.6	31.7
20–49	3.9	10.6	18.8	35.7
≥50	4.1	16.2	15.1	34.8

* SF < 15μg/L; ** ZnPP > 40 umol/mol heme; *** ND = not done.

The prevalence of ID based on ZnPP level of >40 μmol/mol heme among various age groups of both genders is also presented in Table 2. For the age group 15 to 19 years old, females had a higher prevalence of elevated ZnPP values than males. Similarly, adult females 20 years old and older had a significantly higher prevalence of elevated ZnPP values than males. The percentages adjudged to be ID by the ZnPP level were thus higher than that observed for the SF level (Table 2), and the ZnPP values gave consistently higher percentages of iron deficiency than did the SF level.

### 3.3. Other Hematological-Related Variables

Anemia may be due to deficiencies of nutrients other than iron (Table 3). To examine if other nutrients (RBC folate, serum retinol, or vitamin B12) were associated with low Hb concentrations, the prevalence of anemia was examined in those whose SF ≥ 15 μg/dL (not ID), but whose RBC folate and vitamins A and B12 levels were above or below the established cutoffs. Thus, in the subgroup of those who had normal iron levels but low vitamin B12 levels, 1.3% of males and 5.6% of females were anemic. In those with normal iron levels (SF ≥ 15 μg/L) but with low vitamin A levels, 2.6% of males and 10.8% of females were anemic. Finally, in those who had low RBC folate levels but ≥SF levels, 2.3% of males and 9.4% of females were anemic. Multiple regression analysis was performed to describe the relationship between Hb concentration and RBC folate and vitamins A and B12 levels. The overall regression equation was not significant (*p* > 0.05). Parameter estimates for vitamins A, B12, and RBC folate were also not significant. The amount of variation in Hb explained by the independent variables was low (R^2^ = 0.003).

**Table 3:** Prevalence of percent anemia in Kuwaiti adult males and females with low RBC folate, vitamins A and B12.

Variable **	Males		Females	
	Mean ± SEM	% Anemic *	Mean ± SEM	% Anemic
RBC Fol	521.4 ± 11.2	2.3	613.7 ± 13.4	9.4
Vitamin A	73.1 ± 2.2	2.6	64.7 ± 3.7	10.8
Vitamin B12	380.0 ± 16.3	1.3	382.3 ± 15.3	5.6

* (SF ≥ 15 μg/dL); ** Units: RBC Fol (ng/mL); Vit A (μg/dL); Vit B12 (pg/mL).

## 4. Discussion

Several studies of anemia in Kuwait have focused on hemoglobinapathies such as sickle cell disease or α- or β-thalassemia [7,6,26]. Studies of nutritional causes of anemia and ID in Kuwait are based on small non-representative samples taken from clinical or hospital-based studies [8,9,10]. There are few recent studies [14,27] of ID and anemia in non-pregnant Kuwaitis. In pregnant women, [15] found an anemia prevalence of 24.1% but 55% of their 465 sample (18 to 47 years old) had depleted iron stores (SF < 12 μg/dL).

The prevalence of anemia and ID did not differ by level of education or income, probably due to the relative homogeneity of Kuwaiti citizens and due to the Government’s provision of a variety of social amenities, including free education and health care to its citizens. Kuwaiti’s food supply is widely available and highly subsidized. Excessive calories are widely consumed, as evidenced by the estimated 40% obesity rate for Kuwait [19].

Our study has provided the first national estimates of anemia and ID in Kuwait. The prevalence of anemia was 3% and 17%, in 20 to 49 years old males and females, respectively. According to the World Health Organization (WHO) classification of public health risk based on prevalence of anemia, Kuwait falls into the “mild” to “moderate” category with a prevalence of anemia between 5%–22% [2].

Serum Ferritin and ZnPP were measured as indicators of ID. Generally, the ZnPP test indicated twice the percentage of ID as did SF. ZnPP is faster and less expensive to perform than the SF test. However, the high rate of false positives among Kuwaitis has made the ZnPP less useful. A previous study have found a high percent of children with elevated ZnPP levels and ascribed it to high blood lead levels [28]. They concluded that “lead poisoning is a serious problem in some areas of Kuwait”. The higher percentage of “iron deficiency” that we found with the ZnPP test may also have been partly due to lead exposure.

The prevalence of anemia and ID (Table 1 and Table 2) in adolescent males (12 to 14 years old) may be due to increases in lean body mass and later menarche onset (~12.8 years old) in females [14]. The prevalence of anemia was higher in older (50 years and older) compared to younger adult Kuwaitis (20 to 49 years old). The reasons for this are unclear, but may be related to contributions to anemia from other causes, such as chronic diseases.

In males and females who were not ID (SF ≥ 15 μg/dL), varying percentages of anemia were found in those who had low vitamin B12 levels, low RBC folate, or low levels of vitamin A (Table 3). This would suggest that iron may not be the only micronutrient associated with observed anemia in this population. Further investigations of their roles in anemia causation are warranted. Studies in populations with multiple nutrient deficiencies however, have not shown a consistent benefit of multivitamin-multimineral supplementation on improving Hb and in reducing anemia [3,29,30] in non-pregnant anemic women.

Low RBC folate and vitamins A and B12 levels could explain a small percentage of the variation in Hb values, as seen in multiple regression modeling. The multiple contributors to anemia found in this study suggested that intervention programs should carefully consider which mix of supplements and other strategies are needed to ameliorate anemia in Kuwaitis. We did not study genetic causes of anemia; however, hemoglobinopathies do not contribute significantly to anemia prevalence in Kuwait [14].

Results from the Kuwait Nutrition Surveillance Program of over 13,000 individuals of both sexes, representing all ages, gave some similar results to those in our study. In that study, the prevalence of anemia was higher in females than males and 18%–32% of children under five years old, 14%–25% of 5 to 9 years old, 12%–25% of adolescent females and 8%–25% of adolescent males were anemic [13].

The lower prevalence of anemia found in our study compared to earlier studies [12,13] may be attributed to a number of factors, including the inclusion of iron in the wheat flour fortification program initiated in 2001. Evaluation of fortification coverage revealed high percentages of Kuwaiti households purchasing and using fortified products; 79% of households purchased fortified bread; while 87% and 84%, respectively, purchased fortified buns and flour [27].

Our results showed a lower prevalence of anemia in the less than 5 years of age and the 5 to11 years old age groups than that found previously. However, the prevalence of anemia in adolescent females and males are similar to that observed nearly a decade ago [13]. The persistence of high levels of anemia and ID in Kuwait demands increased public health attention. Our data showed a lower prevalence of anemia in school children compared to that found in Saudi Arabia, where anemia was found in 20.5% of over 800 children [31]. Similar to our study, [32] found that age, but not other socioeconomic status (SES) indicators, was a significant independent predictor of both iron depletion and iron deficiency anemia.

## 5. Conclusions

In conclusion, this study could provide new epidemiological data on anemia and ID for Kuwait. It showed that the prevalence of anemia varied by age and gender, but not socioeconomic status. The prevalence of anemia in Kuwait is lower than that in several of its Arabian Gulf neighbors, but still higher than that found in national studies on Americans. These data can serve as a baseline for future studies and interventions and can also be used for comparison with other countries and regions of the world.
